# Experimental Investigation on the Influencing Factors of Compressive Strength of Foamed Lightweight Material Utilizing Completely Decomposed Granite

**DOI:** 10.3390/ma15031060

**Published:** 2022-01-29

**Authors:** Pei Tai, Zhongkui Chen, Zhaofeng Li, Rui Chen, Hu Lu, Yongjia Li

**Affiliations:** 1 School of Civil and Environmental Engineering, Harbin Institute of Technology, Shenzhen 518055, China; taipei@hit.edu.cn (P.T.); lizf09@foxmail.com (Z.L.); cechenrui@hit.edu.cn (R.C.); 18566688842@163.com (Y.L.); 2 Department of Technical Research and Development, Shenzhen Yanzhi Science and Technology Co., Ltd., Shenzhen 518101, China; 3 School of Construction and Environmental Engineering, Shenzhen Polytechnic, Shenzhen 518055, China; lvhu1212@163.com

**Keywords:** completely decomposed granite (CDG), foamed lightweight soil, Taguchi method, compressive strength, water absorption

## Abstract

The utilization of construction waste soil to produce foamed concrete together with cement and a foaming agent is a promising method for waste recycling. Completely decomposed granite (CDG), which is widely available in southern China, was selected as a typical construction waste soil in foamed material production. The Taguchi method was applied to study the influence of various parameters on compressive strength, including cement dosage, CDG dosage, water to solid materials ratio (W/M), fine particles content, and gravel particles content. Analysis of variance (ANOVA) on a CDG-based sample showed that all factors have significant effects on compressive strength and the most effective parameter was cement dosage, followed in sequence by CDG dosage, W/M, gravel particles content, and fine particles content. However, only cement dosage and W/M influence the internal structure significantly during water/vacuum-immersion tests. The relationship between micro-pore structure and compressive strength suggested that with the decrease of open porosity, the compressive strength showed an increasing trend. This study reveals the possibility of CDG as a raw material for foamed lightweight soil and provides a technical reference of production procedure.

## 1. Introduction

With the rapid development of urban construction (especially underground projects) in China, a large amount of construction waste soils has been produced. For instance, annually over 30 million m^3^ of construction waste soils have been produced solely in Shenzhen city. Completely decomposed granite (CDG) is the main component of construction waste soils in southern China, which is also a type of residual soil found in many other parts of the world. Landfilling is one of the most common ways of waste soil disposal, which occupies massive land resources and far exceeds the landfill capacity, sometimes even creating a threat to the urban water safety [1,2]. To tackle this problem, recycling of construction waste soils as new construction materials has attracted more attention in recent years [3,4,5,6], Foamed lightweight soil which is made of foam, cement-based cementitious material, water, aggregates and additive in particular proportions, is one of the emerging methods to address the issue above. Because of the light weight and high-strength characteristics, it is widely applied in areas such as subgrade backfill, bridge backfill, compensated foundations, anti-freezing foundations, mine backfill and so on.

Many studies have been carried out to study the mechanical behaviour of foamed lightweight soil. Ramamurthy et al. [7] studied the effect of foam content and water-cement ratio on the density and compressive strength of foamed lightweight soil. Their results indicated that there was an optimal foam content in the range of 20~50% (volume percentage). Furthermore, high ratio of water-cement leaded to a thin slurry and hence the failure of foam formation, whereas lower ratio of water-cement results in a thick slurry and breakage of the foam. Tam et al. [8] found that addition of micro foam will greatly reduce the wet density and the shrinkage value when the wet density is high. Nambiar et al. [9] pointed out that addition of fine aggregate increases the dry density and improves the compressive strength of the foamed concrete. Moreover, Kearsley et al. [10] found that increasing content of fine aggregate decreases pore size and improves the uniformity of pore distribution in their studies of foamed materials. Despite the good mechanical performance, the increasing cost of raw material, such as river sand and gravel, has become the main limitation to the application of the lightweight soil technique, due to the high requirement of environmental protection. Therefore, it is natural that industries attempt to replace conventional aggregates in foam concrete, such as recycled glass aggregate, expanded shale aggregate, lime, and stone powder [11]. Moreover, some other recycled materials with high activity like silica fume, fly ash and ground fine slag powder, were also used to partially substitute cement in foam concrete, and the results showed that the compressive strength can be improved by up to 25% with proper mixing proportions [10]. It can be concluded that there is a great potential for adopting recycled solids to produce foamed lightweight material, however, to the best of the authors’ knowledge, there is no research regarding utilizing construction waste soil to replace aggregates or cement in foamed lightweight soil.

In this study, completely decomposed granite (CDG) was used to produce foamed lightweight materials. The objective is to quantify the influencing factors of the compressive strength of the CDG-based foamed lightweight material. To obtain the optimal combination of parameters considered, different controlling influential parameters were assessed using Taguchi method. Experimental results were analyzed by analysis of variance (ANOVA) to investigate the significance of these parameters.

## 2. Materials and Methods

### 2.1. Waste Residue Soil

The CDG soil used in this study was collected from a local excavation site of Shenzhen, China. Basic properties of the CDG including Atterberg limits, optimal water content, and maximum dry density were determined according to ASTM standard and the results are summarized in Table 1, more details of parameters of this soil can also be found elsewhere [12,13,14,15]. According to the unified soil classification system (USCS), the soil can be classified as silty sand. The soil was, firstly, cleaned with water and then oven-dried at 105 °C for 24 h before making lightweight soil. This operation is to eliminate any influence from possible organic components.

### 2.2. Cement and Agent

The cementitious material used in this study was *conch* 42.5 ordinary Portland cement with a 3-day compressive strength of 27.4 MPa and 28-day compressive strength of 45 MPa. The mineralogical characteristic of the cement and main physical properties are summarized in Table 1. Sodium sulfate and triethanolamine were used as early strength agent in the experiments. The mass ratio of sodium sulfate to cement and the ratio of triethanolamine to cement were 2.5% and 0.1%, respectively.

To obtain foamed lightweight soil, a plant protein-based foaming agent was introduced in the preparation process. Trial test results suggested that, when the ratio of agent to water is 1:35 by volume and the wet density of the mixture is 40 kg/m^3^, the film inside is elastic, which can be immediately restored to its original state after being pressed by external forces. These characteristics make it form a spherical seal in the CDG-cement slurry.

### 2.3. Specimen Preparation

**Figure 1:**
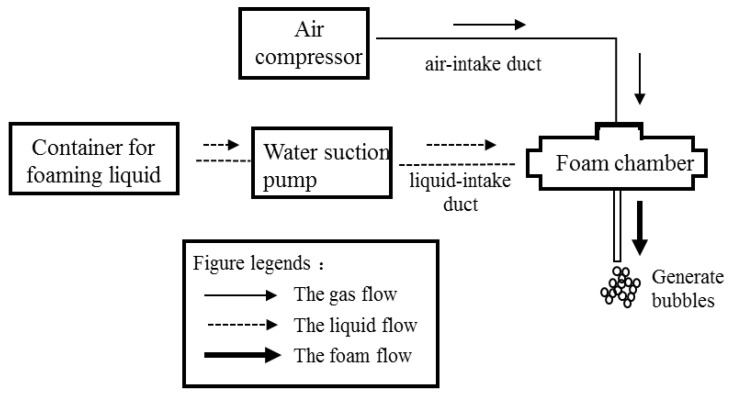
Flow chart of foam preparation.

The foaming process was carried out with high-pressure foaming technology, where an air compressor was used to generate high air pressure. Air was introduced from an air-intake duct into a foam chamber. Meanwhile, the foaming liquid was sucked into the chamber by an electric water suction pump through a suction pipe. The gas-liquid phase met and mixed evenly in the chamber. The overview of the foaming equipment and the foaming principle is shown in Figure 1.

To prepare the foamed lightweight soil, the original soil and cement with a certain proportion was firstly mixed thoroughly using a concrete mixer, then the prepared bubble as shown in Figure 1 was fed into this mixer as well, the whole mixing process would not be stopped until the target density was reached. Afterwards, the prefabricated foaming agent solution was added into the mixture to form a uniform and stable fresh sample. After mixing, the fresh sample was immediately placed into plastic 100 mm cube molds and vibrated manually. After the sample molding, the specimens were released from the molds, sealed with a plastic bag, and kept at 20 ± 2 °C until being tested. The mold releasing time is 24 h.

### 2.4. Test Program

The Taguchi method was utilized to study the CDG-based foamed concrete. This method is a series of techniques based on concepts from the design of experiments [16]. It is a systematic method to reveal the optimal combinations of factors by a small number of experimental runs. Moreover, it can eliminate problems caused by data scattering or system deterioration [17].

A standard L_16_ (4^5^) orthogonal array with five parameters, including cement dosage, CDG dosage, water to solid materials ratio, fine particles content, and gravel particles content was selected here. The detailed levels for each factor and the complete description of CDG-based foamed concrete mix are presented in Table 2. The testing program is shown in Table 3. The range of content of fine and gravel particles was determined by the statistical analysis of nearly 100 soil samples, including soil samples in Songzi pit, Xiaomeisha, Xili and other places in Shenzhen, while CDG with the dosage of 400–700 kg/m^3^ was used to replace coarse and fine aggregates. The cement consumption range was 200–350 kg/m^3^. Compared with the water–cement ratio, the water–solid material ratio has the advantage of reflecting the effect of the change of dry material consumption on water. In terms of traditional concrete, the water consumption largely depends on the amount of cement, however, in the foamed lightweight soil, the fine particles of CDG would absorb a considerable part of water as well, therefore, the water–cement ratio cannot show the water needed (or the degree of hydration) in the production of lightweight soil anymore. Instead, the water–solid ratio, which takes all the solid amount into account, is a much more appropriate choice to show the water consumption. Thus, the water–solid material ratio of 0.35–0.5 was utilized with the consideration that a large amount of water was needed to moisten CDG particles.

### 2.5. Test Procedure

A compressive strength test, wet density test and fluidity test at each concrete mixture were conducted in accordance with the technical specification for foamed mixture lightweight soil-filling engineering [18]. A compressive strength test for each concrete mixture was carried out at the ages of 3, 7 and 28 days of curing using a universal testing machine (model: DNS 300). With a constant loading rate of 2 kN/s, the compressive strength was calculated as the ratio of the failure load to the bearing area of the specimen. The mean of three samples was considered as the final compressive strength for each concrete mixture. In order to obtain the internal pore structure characteristics of CDG-based foamed concrete, both a water-immersion method and vacuum-immersion method was carried out. The vacuum-immersion method can greatly shorten the soaking time and relieve the influence of ion corrosion. Furthermore, it can avoid the phenomenon of secondary hydration caused by the drying method.

The specimens were firstly placed into an electric blast drying oven and kept for 24 h at 60 ± 5 °C, 80 ± 5 °C, respectively. After that, they were dried at and 105 ± 5 °C until their mass became constant (*m*_0_). After temperature of the specimens dropped down to the room temperature, half of them were directly immersed in water for water absorption test. The rest of the specimens were, firstly, vacuumed and then de-aired water was added into the vacuum chamber. After immersion for 24 h, the specimens were wrapped using plastic films under water-immersed conditions. The total mass of each sample and plastic film (*m*_1_) was obtained. The volumetric water absorption *ω* was calculated using Equation (1). The arithmetic mean value of the three samples was taken as the final water absorption of the foamed concrete:

(1) $$\omega =\frac{m_{1}-m_{0}-m_{2}}{\rho V}$$

where *ω* is volumetric water absorption (%), *m*_0_ is the mass of the sample when dried to a constant mass (g), *m*_1_ is the mass of sample after water absorption (g), *m*_2_ is the mass of plastic wrap for wrapping sample (g), *ρ* is the density of water, and *V* is the volume of the sample (cm^3^).

### 2.6. Statistical Analysis

Analysis of variance (ANOVA) with the aim of appraising the significance of parameters on quality characteristics was performed. The percentage of contribution can be determined by calculating the sum of square of each parameter over the total sum of square. Also, the F-test can show the contribution. In the F-test, F-value is used to evaluate the differences between compared groups. The larger the value of F-value is, the greater the influence of explanatory variables on the explained variables.

Apart from ANOVA, the signal to noise ratio (S/N) was applied in the experiments with replications rather than the means because this ratio indicates the variation among the responses. The greater this ratio, the smaller the variance around the target value [19]. In this study, the S/N ratio was used to analyze the experimental results. In Taguchi method, three types of S/N were defined, which are “bigger is better”, “nominal is the best” and “smaller is better”. As for compressive strength, the aim is to maximize the response. Therefore, the S/N of “bigger is better” was used. The equation is shown in Equation (2):

(2) S/N=−10log(MSD)

(3) $$MSD=\left(y_{1}^{2}+y_{2}^{2}+y_{3}^{2}+\ldots \right)/n$$

where *MSD* is the mean squared deviation from the target experimental value; *y_1_*, *y_2_*, *y_3_* denotes the results of experiments; and *n* denotes the number of repetitions.

## 3. Results and Discussions

### 3.1. Compressive Strength Characteristics

The compressive strength of CDG-based foamed lightweight soil is presented in Table 4. It should be noted that the samples of group 7 and group 12 showed severe collapse, which did not meet the test regulation. Therefore, the compressive strengths of group 7 and group 12 were not measured. It can be observed that the compressive strengths of the CDG based foamed concrete specimen are in the range of 0.28–1.56 MPa for a curing of 3 days, 0.36–2.08 MPa for a curing of 7 days, and 0.438–2.70 MPa for a curing of 28 days. The compressive strength of the specimen shows an ascending trend with the increasing curing time.

Based on the results of ANOVA of 28-day foamed concrete specimens as shown in Table 5, all the five explanatory variables have significant effects on the compressive strength of specimens (*p* < 0.05). Among the five parameters, the most effective was the cement dosage accounting for the contribution of 58.53%. The least effective one was proportion of fine content (2.44%) followed in sequence by the proportion of gravel grains (8.93%), water-solid material ratio (10.31%) and CDG dosage (18.82%).

The S/N ratios of each factor presented in Table 6 were used for further analyses. Table 6 shows the average value of S/N ratio for each level of each parameter. It is explained that the higher values of the S/N ratio, the better the control factor combinations minimizing the effects of the noise factors [19].

**Figure 2:**
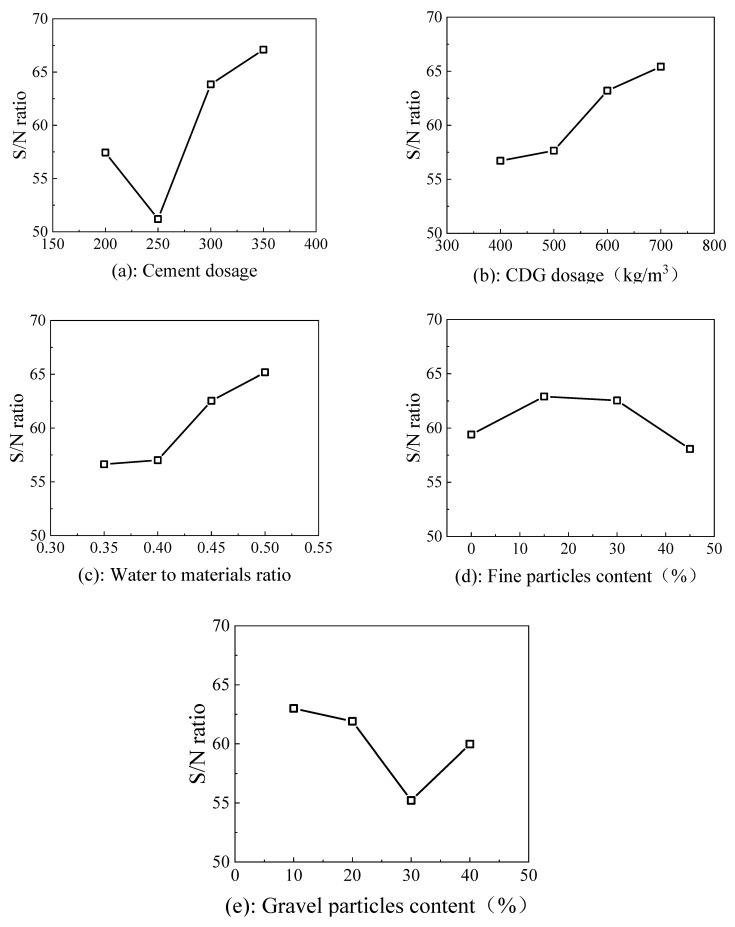
Factor effect diagrams of S/N ratios from compressive strength experiments after 28 days (**a**) cement dosage; (**b**) CDG dosage; (**c**) water to materials ratio; (**d**) fine particles content; (**e**) gravel particles content.

The main effects for compressive strength acquired by plotting the S/N ratio against the corresponding level of the parameters are featured in Figure 2. The main effect is an indication of the performance of each factor on a given process. Studying the main effect of each parameter characterizes the general trend of the factor influence towards the process [20]. As the cement dosage increases, S/N ratio firstly decreases and then increases (see Figure 2a). The reason may be that as the cement dosage is lower, contribution of the hydration reaction for the strength is negligible and even degrades the strength. As the cement dosage further increases, the hydration reaction starts to contribute the higher strength.

It can be observed that with the increase of CDG dosage and water–solid material ratio, the compressive strength presents an increasing trend (see Figure 2b,c). By contrast with conventional concrete, the strength of foamed lightweight material mainly depends on the bonding interface and pore structure. The bonding interface of the mixture is mainly formed by cement and aggregate. Increasing the cement and CDG dosage, together with a higher water–solid material ratio which provides enough water to hydration reaction, both enhances the strength of the “pore wall” and improves the overall strength.

For the increase of the content of fine particles, the compressive strength firstly increased and then decreased (see Figure 2d). In contrast, for the increase of gravel particles content, the compressive strength decreased firstly and then increased (see Figure 2e). The reason is that, with the increase of the proportion of fine particles in specimens, the diameter and uniformity of the internal pore are improved [21]. On the other hand, a large number of fine particles fill the capillary pore gap and play a densifying role, thus contributing to the improvement of compressive strength. However, as the proportion of fine particles continues to increase, the coarse particles act as the skeleton in the foamed concrete decrease, and the strength mainly depends on the bonding of cement. Moreover, due to the large specific surface area of fine particles, the wetting surface needs more water when the coarse particles content decreases (fine particles content increases), which results in an incomplete hydration, leading to a weak strength of the specimens.

From the main effect plots, the level of each factor that corresponds to the highest compressive strength can also be found, which is shown in Table 7. It needs to be noted that the actual optimal values are not exactly those that appeared in the current experiment scheme. Further work is still needed to predict the optimal condition through mathematical modeling and extra experiments.

### 3.2. Water Absorption Characteristics

**Figure 3:**
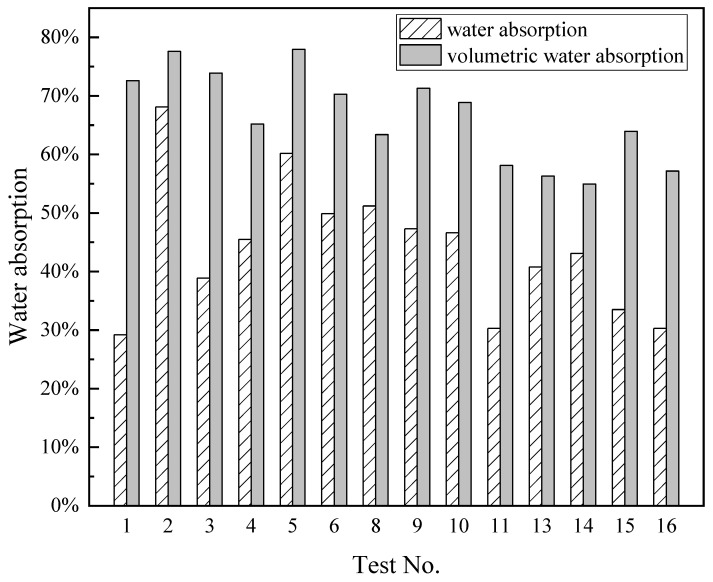
Comparison of water absorption at different test series.

**Figure 4:**
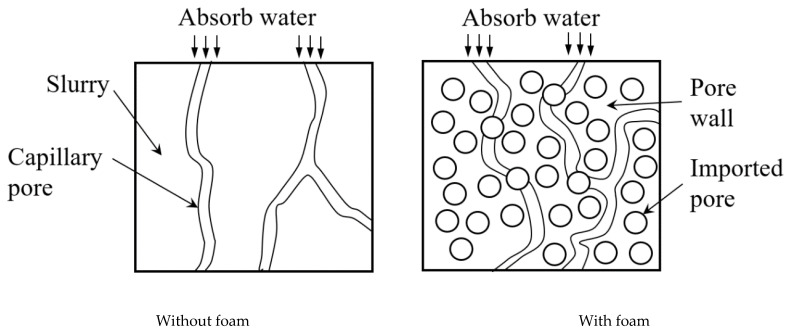
Schematic diagram of pore characteristics with and without foam.

The pore structure of foamed lightweight material is crucial to its compressive strength. According to different closed complete conditions, the internal pores can be divided into closed pores and open pores. The latter can be penetrated by water which affects the strength. Therefore, the water-immersion method and vacuum-immersion were undertaken to study the influence of open pores on the strength of foamed lightweight soil. Figure 3 shows the comparison of water absorption at different test series. Since the pre-determined wet density and fluidity of these test series are different, the water absorption varies. For all the test series, water absorption is lower than the volumetric water absorption. This also demonstrated that results obtained by vacuum-immersion method were much higher than that by the water-immersion method at the same test time, indicating that the internal pore structure of foamed material greatly hindered the absorption of water. The reason is that the internal migration of water in the foamed material is mainly through the capillary in the pore wall, which is driven by capillary absorption. The porosity formed by artificially introducing foam into the slurry increases the curvature and length of the capillary along which the water travels as shown in Figure 4, acting as a barrier to water migration. In the water-immersion method, although the capillary is connected and water can replace the original air in these pores, the artificially introduced foam bubbles are separated by the pore wall and are independent of each other; therefore, water can only enter the pore through a defect in the pore wall and barely displace the air inside completely, which hinders the absorption of water [22]. However, when undertaking the vacuum-immersion test, the air in the pores is drawn out through the capillary, and the resistance of the gas to absorption of water disappears. Water can enter through the capillary pores from the defects on the wall of the pores and fill the bubbles, making the test result higher than that of water immersion [22].

The vacuum-immersion test can largely reflect the real open pore volume of the foamed material samples as the volumetric water absorption directly, which is essential to the durability of foamed lightweight soil in the humid environment (a high water absorption leads to a low durability). In this regard, the analysis of variance (ANOVA) was employed again with the aim of appraising the significance of parameters on the volumetric water absorption, as listed in Table 8. It shows that the most efficacious parameter is the cement dosage for a contribution of 41.22%. The least effective one was CDG dosage (0.34%) followed by proportion of fine grains (0.68%), proportion of gravel content (2.03%) and the water–solid material ratio (6.76%). It also shows that not all factors have a significant effect on the explained variable. Only cement content and the water–solid material ratio have appreciable impact on the volumetric water absorption (*p* < 0.05).

**Figure 5:**
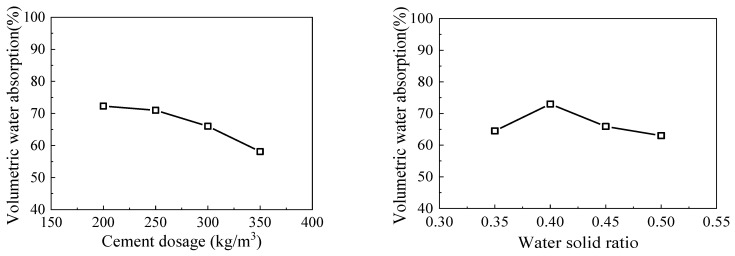
Main effect plots of significant factors to volumetric water absorption.

The main effect plots of significant factors on volumetric water absorption are featured in Figure 5. With the increase of cement dosage, the volumetric water absorption decreased. The main reason is that in the process of sample molding, cement particles adhering in an artificial preformed foam gradually turned into binding material and took the place of liquid film of the preformed foam. The dosage of cement increasing, the hydration product formed around the air-foam become thicker. The product can provide strength and the more the hydration products, the less likely the air-foam surface is to form defects. Consequently, the water absorbing through the capillary cannot infiltrate the pore, which makes the volumetric water absorption decrease.

The volumetric water absorption was increasing first and then decreasing with the increase of the water–solid material ratio. The reason is that a lower water–solid material ratio results in a higher slurry consistency. This causes non-uniform distribution of foams added in the mixing process in the slurry. However, as the water–solid material ratio kept increasing, the slurry flow value increases and hence causes the foams to deform easily. When considering the durability, the cement content and water–solid material ratio should be the main factors considered.

### 3.3. Influence of Open Porosity on Compressive Strength

**Figure 6:**
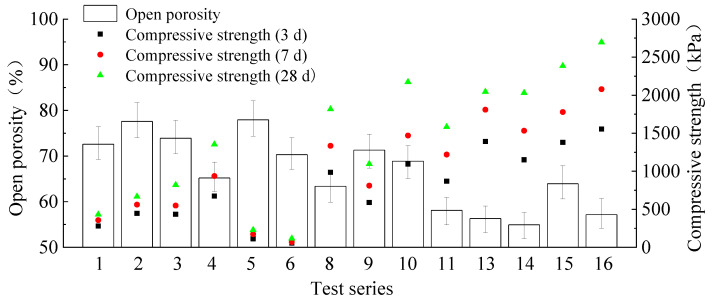
Open porosity and compressive strength at various curing ages.

Figure 6 shows the relationship between compressive strength and open porosity of the vacuum-immersion test. It can be seen that, with the decrease of open porosity, the compressive strengths at all ages show an overall rising trend. This indicated that open porosity is inversely proportional to the compressive strength at each age. The reason is that in the process of slurry forming, the cement particles adhering to the artificial introduction of bubble liquid film surface slowly generate gelatinous substances, and gradually replace the liquid film in the process of liquid film thinning and disappearance. Thus, the hydration products form a solid cement stone pore wall surrounding the air. Defects in the stomatal walls of the open pores allow infiltration by water absorbed by capillaries. The higher the stomatal wall strength is, the less likely it is to form defects on stomatal surface, and the lower the open porosity. Meanwhile, the improvement of stomatal wall strength increases the overall strength of the sample.

## 4. Conclusions

This study explores the possibility of waste soil (CDG) as a raw material to produce foamed lightweight soil and provides a technical reference. The effect of cement dosage, CDG dosage, water–materials ratio, fine particles content, and gravel particles content on the compressive strength of the CDG-based foamed lightweight soils was investigated experimentally. Overall, the test results suggest that it is a promising way to recycle CDG waste soil as a main component of lightweight soil. The detailed conclusions are as follows:

(a) In unit volume, the compressive strength of foamed lightweight soil increases with curing period. The compressive strength of foamed samples increases as the cement and CDG content as well as water–solid material ratio increase. With the increase of the proportion of fine grains, the compressive strength increased first and then decreased, while the effect of gravel content showed an opposite trend.
(b) According to ANOVA, all the explanatory variables involved in the orthogonal test had significant effect on the 28th day of the compressive strength test of the samples. The result also indicated that the most efficacious parameter is the cement dosage for a contribution of 41.22%. The least effective one was CDG dosage (0.34%) followed by the proportion of fine grains (0.68%), proportion of gravel content (2.03%) and water–solid material ratio (6.76%).
(c) With the decrease of the open porosity, the compressive strength of the foamed concrete showed an increasing trend. This is because the higher the pore wall strength is, the less likely the pore surface will form defects, and the lower the open porosity will be. Meanwhile, the higher the pore wall strength also contributed to a higher overall strength.

It is also necessary to mention that this study is just a preliminary laboratory attempt at producing CDG-based foamed lightweight soil. There is still some future work to be done before field applications, such as determining the interaction among processing parameters, the precise cost effectiveness compared to traditional methods, and so on.

## Figures and Tables

**Table 1:** Properties of testing materials.

Physical Properties of CDG				
Liquid Limit %	Plastic Limit %	Plasticity Index	Optimal Water Content %	Maximum Dry Density g/cm^3^
36.3	24.1	12.2	19	1.66
**Properties of cement P.O 42.5**				
**Composition (%)**	Cl^−^	0.012		
	fly ash	13		
	CaCO_3_	15		
	CaSO_4_·H_2_O	5.14		
	MgO	1.25		
	SO_3_	2.01		
**Properties**	Loss on ignition	≤5.0%		
	Specific surface area: m^2^/kg	357		
	Initial setting time: min	203		
	Final setting time: min	250		
	Flexural strength: MPa	5.9 (3 d)		
		7.7 (28 d)		
	Compressive strength: MPa	27.4 (3 d)		
		45 (28 d)		

**Table 2:** The levels for each factor in experiment design (Taguchi method).

Factors	Cement (kg/m^3^)	CDG (kg/m^3^)	Water to Solid Materials Ratio	Fine Content (%)	Gravel Content (%)
Level 1	200	400	0.35	0	10
Level 2	250	500	0.40	15	20
Level 3	300	600	0.45	30	30
Level 4	350	700	0.50	45	40

**Table 3:** Test program (L_16_ orthogonal array).

Test No.	Factors				
	A	B	C	D	E
	Cement (kg/m^3^)	CDG (kg/m^3^)	Water-Material Ratio	Fine Content	Gravel Content
1	1 (200)	1 (400)	1 (0.35)	1 (0%)	1 (10%)
2		2 (500)	2 (0.40)	2 (15%)	2 (20%)
3		3 (600)	3 (0.45)	3 (30%)	3 (30%)
4		4 (700)	4 (0.50)	4 (45%)	4 (40%)
5	2 (250)	1 (400)	2 (0.40)	3 (30%)	4 (40%)
6		2 (500)	1 (0.35)	4 (45%)	3 (30%)
7		3 (600)	4 (0.50)	1 (0%)	2 (20%)
8		4 (700)	3 (0.45)	2 (15%)	1 (10%)
9	3 (300)	1 (400)	3 (0.45)	4 (45%)	2 (20%)
10		2 (500)	4 (0.50)	3 (30%)	1 (10%)
11		3 (600)	1 (0.35)	2 (15%)	4 (40%)
12		4 (700)	2 (0.40)	1 (0%)	3 (30%)
13	4 (350)	1 (400)	4 (0.50)	2 (15%)	3 (30%)
14		2 (500)	3 (0.45)	1 (0%)	4 (40%)
15		3 (600)	2 (0.40)	4 (45%)	1 (10%)
16		4 (700)	1 (0.35)	3 (30%)	2 (20%)

**Table 4:** Results of orthogonal tests for compressive strength of CDG-based foamed lightweight soil.

Test No.	Pre-Determined Wet Density (kg/m^3^)	Fluidity (mm)	Compressive Strength (kPa)		
			3 d	7 d	28 d
1	810	289	280	357	433
2	980	270	447	562	668
3	1160	264	435	549	820
4	1350	285	675	937	1354
5	910	184	113	173	229
6	1013	165	57	69	117
7	1275	--	--	--	--
8	1378	394	986	1335	1817
9	1015	192	590	811	1097
10	1200	266	1093	1470	2174
11	1215	194	870	1220	1584
12	1400	--	--	--	--
13	1125	273	1392	1809	2045
14	1233	394	1151	1533	2030
15	1330	176	1379	1779	2385
16	1418	177	1556	2079	2696

Note: “--” means no results due to severe collapse before compression tests.

**Table 5:** Analysis of variance (ANOVA) for 28-day compressive strength.

Source of Variation	F-Value	Contribution (%)
Cement (kg/m^3^)	1204.252	58.53
CDG (kg/m^3^)	90.57	18.82
Water to materials ratio	48.91	10.31
Fine particles content (%)	26.87	2.44
Gravel particles content (%)	30.37	8.93
Error	-	0.97
Total		100.00

**Table 6:** The signal-to-noise (S/N) ratio values for each level of the parameters.

Factor	Level 1	Level 2	Level 3	Level 4
Cement (kg/m^3^)	57.44	51.20	63.84	67.10
CDG (kg/m^3^)	56.71	57.65	63.20	65.42
Water to materials ratio	56.64	57.02	62.54	65.18
Fine content (%)	59.40	62.91	62.54	58.07
Gravel content (%)	63.00	61.91	55.22	59.98

**Table 7:** Optimal value leading to highest compressive strength.

Factors	Level	Value
Cement (kg/m^3^)	4	350
CDG (kg/m^3^)	4	700
Water to materials ratio	4	0.5
Fine content (%)	2	15
Gravel content (%)	1	10

**Table 8:** ANOVA for volumetric water absorption.

Source of Variation	F-Value	Contribution (%)
Cement (kg/m^3^)	61.899	41.22
CDG (kg/m^3^)	0.542	0.34
Water to materials ratio	5.040	6.76
Fine content (%)	0.787	0.68
Gravel content (%)	3.030	2.03
Error		18.57
total		100

## Data Availability

Not applicable.
